# Assessing farmers’ willingness to pay for FMD vaccines and factors influencing payment: a contingent valuation study in central Oromia, Ethiopia

**DOI:** 10.1186/s12917-024-04169-7

**Published:** 2024-07-15

**Authors:** Misgana Lemi Layessa, Endrias Zewdu Gebremedhin, Edilu Jorga Sarba, Wakuma Mitiku Bune

**Affiliations:** 1 West Shewa Zone, Ambo District Veterinary Clinic Office, Ambo, Ethiopia; 2 School of Veterinary Medicine, Ambo University, P.O. B 19, Ambo, Ethiopia

**Keywords:** Central Oromia, Foot and mouth disease, Vaccine, Willingness to pay

## Abstract

**Background:**

Foot and mouth disease is a contagious, transboundary, and economically devastating viral disease of cloven-hoofed animals. The disease can cause many consequences, including decreased productivity, limited market access, and elimination of flocks or herds. This study aimed to assess farmers’ willingness to pay (WTP) for foot and mouth disease (FMD) vaccines and identify factors influencing their WTP. A cross-sectional questionnaire survey was conducted on 396 randomly selected livestock-owning farmers from three districts in the central Oromia region (Ambo, Dendi, and Holeta districts. The study utilized the contingent valuation method, specifically employing dichotomous choice bids with double bounds, to evaluate the willingness to pay (WTP) for the FMD vaccine. Mean WTP was assessed using interval regression, and influential factors were identified.

**Results:**

The study revealed that the farmer’s mean willingness to pay for a hypothetical foot and mouth disease vaccine was 37.5 Ethiopian Birr (ETB) [95% confidence interval [CI]: 34.5 40.58] in all data, while it was 23.84 (95% CI: 21.47–26.28) in the mixed farming system and 64.87 Ethiopian Birr (95% CI: 58.68 71.15) in the market-oriented farming system. We identified main livelihood, management system, sales income, breed, keeping animals for profit, and foot and mouth disease impact perception score as significant variables (*p* ≤ 0.05) determining the farmers’ WTP for the FMD vaccine.

**Conclusion:**

Farmers demonstrated a high computed willingness to pay, which can be considered an advantage in the foot and mouth disease vaccination program in central Oromia. Therefore, it is necessary to ensure sufficient vaccine supply services to meet the high demand revealed.

**Supplementary Information:**

The online version contains supplementary material available at 10.1186/s12917-024-04169-7.

## Background

Foot and mouth disease (FMD) is a contagious, transboundary, and economically devastating viral disease of cloven-hoofed animals, including domestic and wildlife species. The disease is caused by foot and mouth disease virus (FMDV), which belongs to the genus *Aphtovirus* and the family Picornaviridae. The virus comprises seven serotypes (A, O, C, Asia1, SAT1, SAT2, and SAT3) with numerous subtypes. Ethiopia has endemic serotypes of Foot-and-Mouth Disease (FMD), including serotypes O, A, SAT 1, and SAT 2, among the seven known serotypes. Clinically, FMD manifests as vesicular eruptions in the oral cavity, foot, and udder, accompanied by symptoms such as fever, lameness, salivation, and anorexia [1].

Foot and mouth disease is the most critical global livestock disease regarding its economic impact. In endemic areas, the economic effects of FMD can be separated into direct and indirect losses, and the annual economic impact ranges from USD 6.5 to 21 billion, covering noticeable production losses and immunization expenses. In contrast, outbreaks in FMD-free countries and zones cost USD 1.5 billion each year [2].

In the crop-livestock mixed farming system of Ethiopia, the economic losses from FMD outbreaks attributable to the disruption of milk production, the loss of draft power, and increased mortality among infected cattle herds were, on average, USD 76. At the same time, it was USD 9.8 per affected head of cattle. In the pastoral system, economic losses of USD 174 and 5.3 per involved herd and head of cattle in the infected herds were estimated. In pastoral systems, the costs associated with these losses are exceptionally high because cattle serve as a crucial source of income and livelihood [3].

Two major approaches are used to control FMD worldwide: vaccination with or without stamping out, as in continental Europe and parts of South America, and intensive surveillance and eradication, as in the case of North America, Scandinavia, and the United Kingdom. In disease-free developed countries, the stamping-out approach is employed to control the incursion of outbreaks. This method involves swiftly detecting the introduction of the disease and culling both infected and in-contact herds to prevent its spread [4]. In Ethiopia, various control methods are employed to manage Foot-and-Mouth Disease (FMD), including the implementation of biosecurity measures. These measures encompass conducting vaccination campaigns, implementing disinfection protocols, ensuring safe disposal of carcasses, raising public awareness and education about FMD, and administering antibiotics to combat secondary bacterial complications [5].

Regular mass vaccinations are commonly used in developing countries to control FMD in endemic regions. However, the use of vaccines that cover multiple serotypes and strains presents challenges due to reduced potency and higher costs compared to single-serotype vaccines [4]. Achieving effective cross-protection among the various serotypes and strains is also difficult [5]. In Ethiopia, FMD outbreaks are widespread across all production systems, particularly in mixed crop-livestock, pastoral, and market-oriented districts. The predominant serotypes causing outbreaks in the country are A, O, SAT 1, and SAT 2 [6, 7]. Serological studies reveal varying levels of seroprevalence (3.4–72.1%), indicating limitations in the effectiveness of the control program, including planned vaccination, high susceptibility of animals, and inadequate biosecurity measures [8, 9].

The National Veterinary Institute (NVI) in Ethiopia manufactures a trivalent Foot-and-Mouth Disease (FMD) vaccine containing inactivated serotypes O, A, and SAT 2 strains, providing six months of immunity. Although the vaccine has an effectiveness falling below the globally indicated 75%, it is recommended for biannual government-administered vaccination [10]. Its relatively high cost (15 ETB/dose) compared to other livestock vaccines discourages small-scale traditional farmers from using it, leading to low vaccination rates and ongoing outbreaks in villages. Despite falling below the global standard, the existing FMD vaccine still holds value in reducing the incidence and severity of clinical disease in vaccinated cattle [10].

Researchers can use contingent valuation to determine the WTP for public goods, such as FMD vaccines. This could be for a vaccine under development and yet not marketed [6] or for existing vaccines that are poorly adapted for a variety of reasons, including price sensitivity [7, 8].

Foot-and-mouth disease outbreaks have resulted in significant financial losses for countries, amounting to millions of USD, due to the restrictions or rejection of livestock products. Despite the importance of vaccination in preventing FMD outbreaks and its economic impact, there is a lack of comprehensive understanding regarding farmers’ willingness to invest in FMD vaccines. This study aimed to assess farmers’ WTP for FMD vaccines and identify factors influencing their WTP.

## Methods

### Study area and population

**Fig. 1:**
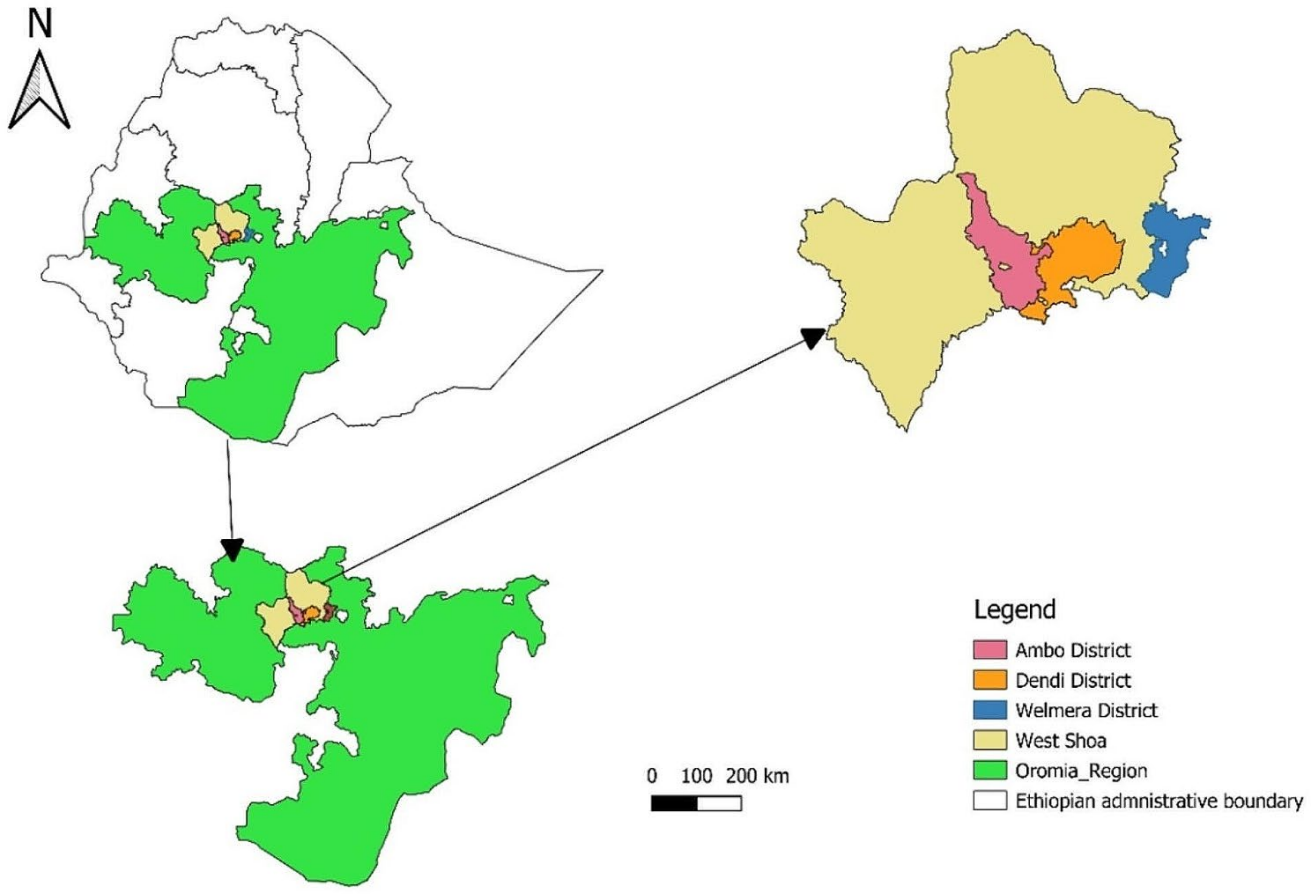
A map showing the study districts in central Oromia, Ethiopia. (Source: QGIS, Version 3.26)

The study was conducted in three districts in the central part of the Oromia region of Ethiopia, namely, the Ambo, Dendi, and Holeta districts (Fig. 1), where the mixed crop-livestock farming system is the basis for the community’s livelihood. There are two main types of production systems practiced in the region: the dominant mixed crop-livestock (MF) production system, which is a subsistence system practiced in rural areas, and a market-oriented (MO) production system, which produces commercial milk in urban and peri-urban areas. The livestock subsector plays an essential role in the livelihood of these areas by providing alternative income sources as a strategy to build emergence to stress and contribute to their food security. Both local and crossbred cattle are raised in the areas.

We conducted the study on the human population residing in the three selected districts. The study subjects were households in the sedentary farming system engaged in livestock rearing as their primary livelihood or occupation.

### Study design

A cross-sectional study was conducted from January to June 2022 to assess farmers’ WTP for the FMD vaccine.

### Sampling technique

Three districts (Ambo, Dendi, and Welmera) and their towns in the West Shoa zone of central Ethiopia were purposefully picked at the outset to ensure the feasibility of the study and accommodate the study area and period. We made this decision based on the observations made among these three districts. Subsequently, we considered six villages from each district, resulting in eighteen villages.

We employed a purposive sampling technique for this investigation but did not conduct a formal sample size calculation. This omission was due to the lack of prior knowledge about the joint distribution of the dependent and independent variables [9]. Since this information was not available before the survey, it was not possible to implement a formal sample size estimate.

We randomly selected farmers from urban streets and rural villages within the designated study areas. We randomly selected 22 households from each of the eighteen chosen villages, utilizing household lists provided by development agents and health extension workers in those villages. As a result, a systematic random sampling method was employed to select households and study 396 participants.

### Contingent valuation method (CVM)

Despite having a price, animal disease vaccines offer public advantages by controlling disease transmission within the community, thereby benefiting the well-being of all animals. Hence, contingent valuation is used to determine the WTP for the FMD vaccine [6]. A survey was designed to produce hypothetical market scenarios that would indicate the value of FMD vaccination using the CVM. Then, the survey respondents were requested to respond to these hypothetical market scenarios.

### The questionnaire survey

The survey questions (32 closed-ended) were initially prepared in English, later translated into Afaan Oromo (the language used in the study area), and personally administered to the selected households by the researchers. A pilot survey was carried out in December 2021 in the community before the actual study to assess the instruments’ suitability and feasibility and to determine respondents’ views of possible vaccine prices to pay. For the pilot survey, 60 individuals (30 from mixed farming and 30 from market-oriented livestock-rearing) were randomly selected from the village using field surveying by asking every person who walks a fixed point along a busy pathway to obtain the first sets of bid prices [10].

In addition, researchers selected WTP sets of bids based on likely vaccine prices available in the market, considering the surveying of bid values in the pilot study. This set of bids obtained from open-ended questionnaire responses was collected as a reference baseline but was not used in the primary analyses. The researchers consulted the National Veterinary Institute (Bishoftu, Ethiopia) to assess the country’s capacity for producing the vaccines included for FMD control. We conducted the final survey from January to June 2022. The respondents who agreed to participate in the study were adults at least 18 years old, heads of household, and currently owned livestock.

The face-to-face survey method was designed to measure potential factors of acceptance of FMD vaccines and WTP for vaccination services. The survey took approximately 30 min for each participant, and households were advised on the animal health and production extension services upon completion of the study to compensate for their time.

The double-bounded dichotomous choice method via closed-ended questionnaire format was used in which the respondents were asked a sequence of two questions about their WTP for FMD vaccine at a specific price so that they used to give a “yes” or “no” response to a single bid question. To enhance the reliability of the contingent valuation questionnaire, researchers included a ‘do not know’ response option and a ‘Yes/No’ response option. To implement this contingent valuation survey effectively, researchers ensured a clear definition of nonmarketed goods (FMD vaccine) and established a credible means of vaccination service provision. They also developed a possible mechanism that facilitated the exchange between the uses of the FMD vaccine and the price of interest. For this purpose, a double-bounded dichotomous questionnaire format was adapted from a previous study in Ethiopia [1].

In this survey of contingent evaluation elicitation questions, researchers utilized the recommended guidelines prepared by the National Oceanic and Atmospheric Administration (NOAA), which consist of two components [11]. These guidelines are general in nature and applicable, particularly in developing countries, which depend mainly on the socioeconomic and institutional aspects existing in the study areas [12]. Following the guidelines outlined, the questionnaire was divided into two components. The questionnaire consisted of two components. The first component included bidding questions directly linked to the WTP for a hypothetical FMD vaccine market price. The second component comprised questions exploring the socioeconomic factors that could impact the farmer’s willingness to pay for the FMD vaccine. Before the two components of the survey, there was a justifying question of whether the respondents knew the disease, which contained a statement of approval for the respondents and some triggering questions to obtain information regarding the respondent’s knowledge about animal health services in addition to FMD disease.

Consequently, this double dichotomous choice bidding format consisted of two stages, where respondents were presented with bid amounts and had the option to indicate their WTP as either ‘yes’ or ‘no’ in response to the proposed hypothetical vaccine prices. The initial bid amounts were distributed uniformly and randomly among respondents who were exposed to the questionnaire. Depending on the respondents’ answer to the initial bid question, the amount of the follow-up bid was increased (premium bid) or decreased (discounted offer) accordingly by a set amount randomized and asked by the respondents, which means that if the response to the initial bid amount was ‘yes,’ the follow-up bid amount would increase by 50%, and if the answer to the initial bid amount was ‘no,’ the follow-up bid amount would decrease by 50% (Table 1). In the first and follow-up stage questions, we included an ‘Undetermined’ alternative for respondents who could not decide between ‘Yes’ or ‘No.’ The initial bid set prices proposed in the first component of this survey contained 20, 40, 60, and 80 Ethiopian Birr (ETB) per dose (1 ETB = 0.0203 USD at the time of the study) (Table 1). Researchers proposed this initial bid price set based on information from an open-ended WTP pilot survey with a price range of 12–100 ETB/annual dose for the same hypothetical vaccine [1]. This price range was nearly comparable to USD 0.4–3 (ETB 12–88) for diverse types of FMD vaccines reported in the literature in other countries [2, 13]. Currently, the Ethiopian government is providing FMD vaccines at a subsidized price of 15 ETB/dose for farmers.

The second component of the questionnaire contained the sociodemographic features and management systems (independent variables), which could affect respondents’ WTP (dependent variable) for a hypothetical FMD vaccine. The key demographic and animal husbandry system variables included were district, sex, age, educational status, household size, main livelihood, tropical livestock unit (TLU) owned, direct livestock income (which is the sum of milk sale and live animal sale), management system, breed, animal isolated for profit, adoption of veterinary service (treatment, vaccination, etc.). FMD impact perception score (perception about FMD impact on livestock), and vaccine knowledge score (knowledge on the use of the vaccine for livestock disease prevention). The present study did not consider all animal-related benefits, such as apiculture, aquaculture, animal byproducts, equines, and pet benefits. Furthermore, we did not consider traits of the vaccine, as they could not be studied using the CVM. Instead, another valuation method called the choice experiment method was employed. We measured the perception of FMD impact and knowledge of vaccines as combined scores of corresponding questions under the two variables. Therefore, we derived the FMD impact perception score from five questions, each with three possible scores, resulting in 15 FMD impact perception scores. Likewise, the knowledge score about livestock vaccines was derived from four vaccine knowledge questions, where a correct response received a score of one and an incorrect response received a score of zero, resulting in a livestock vaccine knowledge score of four [1].

**Table 1:** Double-bounded dichotomous questionnaire bidding structure

Initial bid (ETB)	Initial bid response	Follow-up bid amount (ETB)
20	No	10
	Yes	30
40	No	20
	Yes	60
60	No	30
	Yes	90
80	No	40
	Yes	120

### Data analysis

The data were imported into Microsoft Excel 2007 and manually verified to ensure accuracy. This involved systematically checking for missing values and outliers. All data analyses were performed using STATA software version 14 (Stata Corp. College Station, TX). Descriptive statistics were employed to analyze and summarize the data using various variables. In the interval regression model, the variables considered for the models were checked first for multicollinearity using the variance inflation factor (VIF). We considered a variance inflation factor (VIF) value above ten to indicate collinearity among variables. Consequently, variables such as breed (*r*=-0.72) and management system (*r*=-0.75) were rejected from the model, and the full models containing all noncollinear variables passed the final analysis, which contained MO and MF together. The model was also developed separately for MO and MF production systems, and the same procedure was followed. The final models were reached by excluding nonsignificant variables with a p-value > 0.05 one at a time until only significant variables were left [14].

The responses to the double-bounded contingent valuation (CV) questions give four possible discrete outcomes: (1) the household was not willing to pay for FMD vaccines even at the discounted price (“no,” “no”) to both bids; (2) the household was not willing to pay for FMD vaccines at the initial price but was willing to buy at the discounted price (“no,” “yes”); (3) the household was willing to pay for FMD vaccines at the initial price but not the increased, premium price (“yes,” “no”); or (4) the household was willing to pay for FMD vaccines at both the initial price and the premium price (“yes,” “yes”). This double-bounded model allows us to place the household’s WTP into one of four intervals: (0, Bl), (Bl, Bi), (Bi, Bh), and (Bh, +). where Bi is the initial bid amount, Bl is the lower follow-up bid amount, and Bh is the higher follow-up bid amount.

We employed interval regression analysis [15] to estimate the farmers’ WTP for the FMD vaccine using the double-bounded dichotomous contingent valuation data collected through the questionnaire. This interval regression model gives three types of censoring (left censored, right censored, and interval censored) where the WTP values lie down. After analyzing all interval data, we expressed the values of the STATA output in terms of mean, standard deviation, standard error, percentiles, model coefficients, 95% confidence interval (CI) of coefficients, and P value. Farmers’ WTP for the FMD vaccine was estimated using the interval data estimation method under the assumption that WTP can be modeled as a linear function of the characteristics of respondents along with a standard error [16].

## Results

### Respondent characteristics

Among the 396 farmers surveyed for the dichotomous choice bidding questions, ten answered ‘Do not know’ either to both bids or one of the bid questions. Two outliers were found due to their high income. These 12 observations were excluded from the analysis, which gave us 384 cleaned observations from the three districts. Hence, the questionnaire was administered to 396 households surveyed, which was reduced to 384 observations.

The average age of the respondents was approximately 43 years old (43.7 for MF and 44.15 for MO), and most were males (94%), with an average household member of 4.7. Almost all respondents mentioned that they know about FMD (locally known as “*Kebena*,*”* which means *feeling cold*). Approximately 94% of respondents in the market-oriented system kept cattle for profit (livestock products and live animal sales), but only approximately 19% of mixed farming respondents kept cattle for profit.

Approximately 94% of the respondents believed that vaccines could prevent livestock diseases, 62% used modern veterinary services, and all had experience using other types of vaccines in their cattle husbandry. Table 2 presents the sociodemographic and cattle husbandry characteristics of the respondents. We used tropical livestock units (TLU) to calculate the total number of different species of livestock kept by respondents. As a result, the average TLU was approximately 12.48 for male farmers (MF) and 9.2 for female farmers (MO) respondents. The average income of respondents from livestock sales in MF and MO was approximately 10,737 ETB and 266,707 ETB, respectively. The mean overall FMD impact perception score out of 15 points was 13.11 for MF and 13.58 for MO system respondents. Similarly, the mean vaccine knowledge score was 2.52 for MF and 3.02 for MO out of 4 points.

**Table 2:** Results General characteristics of respondents in Ambo, Dendi, and Welmera districts of Oromia, Ethiopia (MF = 241, MO = 143, *n* = 384)

Variables	*n* (%)	MF (%)	MO (%)
**Sex**	**384 (100)**	**241 (100)**	**143 (100)**
Male	362 (94.30)	233 (96.68)	129 (90.21)
Female	22 (5.70)	8 (3.32)	14 (9.79)
**Age**	**384 (100)**	**241 (100)**	**143 (100)**
18–30 Years	23 (5.98)	15 (6.22)	8 (5.68)
31–50 Years	292 (76.04)	181(75.10)	111 (77.62)
> 50 years	69 (17.97)	45 (18.67)	24 (16.78)
**Educational status**	**384 (100)**	**241 (100)**	**143 (100)**
Illiterate	19 (5.00)	15 (6.22)	4 (2.79)
Primary	278 (72.40)	196 (81.33)	82 (57.34)
Secondary and above	87 (22.60)	30 (12.45)	57 (39.86)
**Household size**	**384 (100)**	**241 (100)**	**143 (100)**
Two members	13 (3.30)	7 (2.9)	6 (4.28)
3–5 members	254 (66.20)	153 (63.48)	101 (70.63)
> 5 members	117 (30.50)	81 (33.61)	36 (25.17)
**Management System**	**384 (100)**	**241 (100)**	**143 (100)**
Intensive	107 (27.80)	9 (3.73)	98 (68.53)
Semi-intensive	39 (10.20)	18 (7.47)	21 (14.68)
Extensive	238 (62.00)	214 (88.80)	24 (16.78)
**Breed**	**384 (100)**	**241 (100)**	**143 (100)**
Exotic	143 (37.20)	25 (10.37)	118 (82.51)
Local & cross	241(62.80)	216 (89.63)	25 (17.48)
**Animals kept for profit**	**384 (100)**	**241 (100)**	**143 (100)**
Yes	174 (45.30)	46 (19.09)	128 (89.51)
No	210 (54.70)	195 (80.91)	15 (10.48)
**Usage of the vet. Services**	**384 (100)**	**241 (100)**	**143 (100)**
Never	10 (2.60)	10 (4.15)	0 (0.00)
Sometimes	136 (35.40)	124 (51.45)	12 (8.39)
Always	238 (62.00)	107 (44.40)	131 (91.61)

Key: MF, mixed farming system MO, market-oriented system, SD, standard deviation

**Fig. 2:**
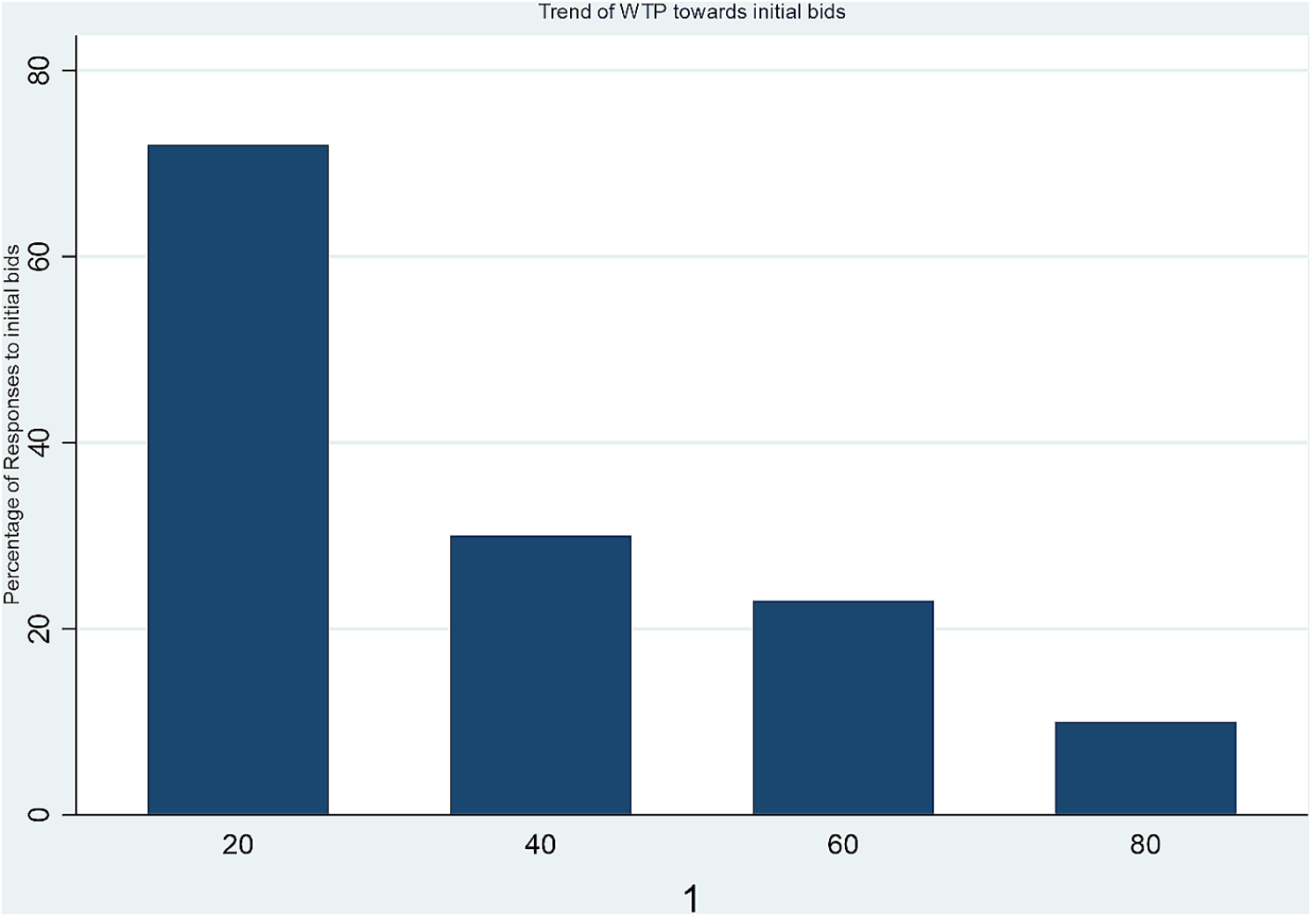
Graph showing the WTP trend regarding the initial bid amount offered to respondents. The “0” and “1” under the graph represent “No” and “Yes” responses toward the initial bids provided to respondents, respectively. The respondents’ WTP (yes response) decreased with the increased bidding amount

The respondents’ WTP for the hypothetical FMD vaccine declined regarding the increase in the bidding amount offered to them, as indicated in Fig. 2. At the same time, the WTP increased as the bidding amounts decreased.

Among the all-initial bids offered for all respondents, 35.20% received ‘Yes’ responses, and 64.8% received ‘No’ answers. The follow-up bids received more ‘Yes’ reactions when the bidding amount was lower. In separate bids, the overall percentage of obedience (yes response) for initial bids of 20 ETB, 40 ETB, 60 ETB, and 80 ETB was 74%, 31%, 24%, and 11%, respectively (Table 3).

**Table 3:** Summary of initial and follow-up bid responses in the double-bounded dichotomous choice in total sample size (*N* = 384)

Initial Bid (ETB)	Initial bid and responses		Follow-up bid and responses		
	Responses	Responses (%)	Follow bid(ETB)	No (%)	Yes (%)
20	No	25 (26)	10	9 (36)	16 (64)
	Yes	72 (74)	30	41(56)	26 (36)
40	No	67 (69)	20	38 (58)	27 (41)
	Yes	30 (31)	60	16 (51)	15 (48)
60	No	73 (76)	30	54 (76)	22 (30)
	Yes	23 (24)	90	11 (44)	14 (56)
80	No	84 (89)	40	52 (62)	31 (37)
	Yes	10 (11)	120	5 (41)	7 (58)
**Total**	**No**	**249 (64.80)**		**153 (61.45)**	**96 (38.55)**
	**Yes**	**135 (35.20)**		**73 (54)**	**62 (46)**

The percentages of WTP (‘yes’ responses) in MF respondents for the proposed bids of 20 ETB, 40 ETB, 60 ETB, and 80 ETB were 61%, 9.2%, 4.8%, and 3.7%, respectively (Table 4).

**Table 4:** Summary of initial and follow-up bid responses in the mixed farming system (MF = 241)

Bid (ETB)	Initial bid responses		Follow-up bid and responses		
	Responses	Responses (%)	Follow bid (ETB)	No (%)	Yes (%)
20	No	23 (39)	10	9 (39.00)	14 (61.00)
	Yes	36 (61)	30	34 (87.20)	5 (12.80)
40	No	59 (90.80)	20	36 (62.00)	22 (38.00)
	Yes	6 (9.20)	60	6 (100)	0 (0.00)
60	No	60 (95.20)	30	49 (92.50)	4 (7.50)
	Yes	3 (4.80)	90	4 (80.00)	1 (20)
80	No	52 (96.30)	40	45 (88.20)	6 (11.80)
	Yes	2 (3.70)	120	2 (50.00)	2 (50.00)
**Total**	**No**	**194 (80.49)**		**143 (59.34)**	**51 (21.16)**
	**Yes**	**47 (19.50)**		**42 (17.43)**	**5 (2.07)**

The percentages of WTP (‘yes’ responses) in market-oriented respondents (MO) for the initial bids of 20, 40, 60, and 80 ETBs were 94.7%, 75%, 60.6%, and 20%, respectively (Table 5).

**Table 5:** Summary of initial and follow-up bid responses in the market-oriented farming system (MO = 143)

Bid (ETB)	Initial bid responses		Follow-up bid and responses		
	Responses	Responses (%)	Follow bid(ETB)	No (%)	Yes (%)
20	No	2 (5.30)	10	0 (0.00)	2 (100.00)
	Yes	36 (94.70)	30	11(30.60)	25 (69.40)
40	No	8 (25.00)	20	2 (28.60)	5 (71.40)
	Yes	24 (75.00)	60	10 (40.00)	15 (60.00)
60	No	13 (39.40)	30	1 (7.10)	13 (92.90)
	Yes	20 (60.60)	90	7 (35.00)	13 (65.00)
80	No	32 (80.00)	40	7 (21.90)	25 (78.10)
	Yes	8 (20.00)	120	3 (37.50)	5 (62.50)
**Total**	**No**	**55 (38.46)**		**10 (6.99)**	**45 (31.47)**
	**Yes**	**88 (61.54)**		**30 (20.98)**	**58 (40.56)**

### WTP for FMD vaccine and significant variables

We obtained the mean estimates of WTP using the interval data method, which distributed the latent WTP between the two bounds (lower and upper bounds). The estimates are directly observed from the model and not derived from other parameters. This is because the estimation procedure effectively involves an interval regression with only an intercept.

According to the interval regression analysis, the average WTP per dose, as determined by the constants of the null models (models without any explanatory variables), was estimated at 37.51 ETB (95% CI: 34.53–40.58%) for all respondents. For MF respondents, the average WTP was 23.84 ETB (95% CI: 21.47–26.28), while for MO respondents, it was 64.87 ETB (95% CI: 58.68–71.15). We obtained these WTP estimates from the model without considering other variables that could affect the WTP for the vaccine. The forecasts from the model are presented in Table 6.

**Table 6:** The WTP (ETB) estimates derived from the interval linear regression models

Production type	Mean	Standard Error	Confidence interval (95%)
Whole Data	37.51	1.52	34.53–40.58
MF system	23.83	1.21	21.47–26.29
MO system	64.88	3.24	58.77–71.25

The interval regression model assesses the factors influencing respondents’ WTP for the FMD vaccine. An assessment for multicollinearity using the variance inflation factor (VIF) showed that all the variables had a VIF of less than 10 with a mean VIF of 1.86; hence, except for the production system (collinear with main livelihood), all the variables are not collinear to each other.

The variables significantly associated with WTP were the main livelihood, keeping animals for profit, and FMD impact of perception score. The variable main livelihood of the farmers significantly negatively influences the farmers’ WTP for vaccination. Age and sex were not significant and were not included in the multivariable model. The remaining variables, the management system (excluded due to collinearity) and adoption of veterinary service, district, education level, and TLU, were found insignificant in the multivariable analysis (*p* > 0.05) in determining farmers’ WTP for the FMD vaccine.

The MF and other respondents were less likely (-26.0 less) to pay for immunization than their MO counterparts. Our model also showed that animals isolated for-profit and FMD impact perception scores had a significant effect on WTP. Accordingly, when the FMD impact perception score increases by one unit, the WTP increases by 1.40 ETB, keeping other variables constant in the interval regression model. Similarly, participants who keep cattle for profit purposes had 16.00 more ETB WTP compared to their counterparts, keeping other variables constant in the interval regression model (Table 7).

**Table 7:** Variables associated with WTP for FMD vaccines (*N* = 384)

Variables	Category	Univariable model			Multivariable model	
		Model coef. (CI: 95%)		*P* -Value	Model coef. (CI: 95%)	*P* Value
District	Ambo	Reference				
	Welmera	13.47 (6.32–20.62)		≤ 0.001		
	Dendi	6.70 (-0.43-13.82)		0.066		
Age	Continous	0.189 (-0.13-0.5145)		0.253		
Sex	Male	Reference				
	Female	9.328 (-3.84-22.49)		0.165		
Educational status	Illiterate	Reference				
	Grade 1–8	9.40 (-3.61-22.42)		0.157		
	Grade 9–12 &above	26.11 (12.11–40.11)		≤ 0.001		
Tropical livestock unit (TLU)	Continuous	2.00 (-1.72- -0.678)		≤ 0.001		
Adoption of veterinary service	Never	Reference				
	Sometimes	8.66 (-8.53-25.85)		0.324		
	Always	31.72 (14.76–48.69)		≤ 0.001		
Production system	Extensive					
	Semi-intesnive	30.89 (22.61–39.17)		≤ 0.001		
	Intensive	41.17 (35.57–46.77)		≤ 0.001		
Main livelihood	MF & Others	Reference			Reference	
	MO Farming	-37.94 (-43.11- -32.77)		≤ 0.001	-26.00(-19.36)-(-32.63)	≤ 0.001
Animals are kept for profit.	No	Reference			Reference	
	Yes	33.69 (28.57–38.82)		≤ 0.001	16.00(9.67–22.33)	≤ 0.001
FMD perception score	Continuous	2.60 (1.15–4.05)	≤ 0.001		1.40 (0.24–2.56)	0.018

Due to their socioeconomic and husbandry differences, we conducted separate interval regressions for the two production systems (MF and MO). Accordingly, there was a significant difference in WTP for the FMD vaccine, which warranted separate analyses. The factors significantly associated with the WTP of the FMD vaccine in MF were sales income from the animals and the management system. Accordingly, the WTP increased by 0.002 points for each one-unit increase in sales income, whereas farmers handling their cattle under an intensive management system had 6.8 ETB more WTP than those in an extensive system. On the other hand, the factor significantly associated with the WTP of the FMD vaccine in MO was the types of cattle breeds kept. As a result, approximately 13.26 ETB increases were observed in farmers handling exotic breeds compared to those handling local cattle and their crosses (Table 8).

**Table 8:** Associations between livestock husbandry variables and WTP among respondents in various production systems

Type of production system	Variable category	Model (Coef.)	(CI: 95%)	*P*- value
**MF production system**				
Sales income	Continuous	0.0002	0.0001–0.0003	≤ 0.001
Management system	Extensive	Reference		
	Semi-intesnisve	14.58	4.53–24.64	0.004
	Intensive	15.44	2.51–28.36	0.019
**MO production system**				
Breed	Local and their crosses	Reference		
	Exotic or cross-only	28.44	16.58–40.30	≤ 0.001

Model (Cons.): denotes the constant value of change in WTP about change in variables

## Discussion

The study found an overall average willingness to pay of 37.5 ETB for a hypothetical FMD vaccine among farmers. The WTP was 23.84 ETB in the mixed farming system and 64.87 ETB in the market-oriented farming system.

In Ethiopia, the utilization of FMD vaccines in all production systems has been limited, primarily because of infrequent vaccination and insufficient awareness regarding the circulating serotypes prior to vaccination. Challenges also arise from the reluctance of livestock producers to vaccinate or bear the cost of vaccination. Jemberu et al. [8] identified farmers’ perceived cost of vaccination (perceived barrier) as the most crucial perception that significantly influenced the intention to implement vaccination with payment [17]. The higher average TLU of 12.48 in the MF system, compared to an average TLU of 9.2 in the MO system, could be attributed to the inclusion of other livestock, such as sheep, goats, oxen, and equines within the MF system. In contrast, the MO system primarily focuses on maintaining fewer productive cows for milk production.

The study discovered that the estimated WTP for the proposed vaccine price (mean = ETB 37.51) exceeded that of the currently available government-produced FMD vaccine in Ethiopia (NVI). This finding was unexpected, as most vaccines, including those for transboundary animal diseases, are typically available at lower prices or free in the study area. The percentage of farmers willing to pay decreased gradually from 74 to 11% as the opening bid values improved from ETB 20 to 80, aligning with the economic principles of market behavior [18].

The obedience rate (percentage of “yes” responses) among all respondents was 74% for an initial bid of 20 ETB, 31% for 40 ETB, 24% for 60 ETB, and 11% for 80 ETB. This indicates that the majority of respondents (74%) are willing or inclined to pay the initial bid of 20 ETB, aligning with the typical demand behavior observed in various commodities when prices fluctuate [19].

The estimated mean WTP for the FMD vaccine computed by the interval regression model from the whole data was ETB 37.51 (95% CI: 34.53–40.58) per year, and this revealed a narrow confidence interval of WTP, which indicates a high level of certainty and shows consistency of this study. This is lower than previous studies on farmers’ WTP for the FMD vaccine in the Amhara region, which was computed as ETB 58.23/dose [1]. The WTP for the FMD vaccine can vary among regions based on various factors. The possible reasons for such a scenario might be the increased risk of FMD, access to information and awareness, previous experience with the vaccine’s effectiveness, income levels, market opportunities, and access to veterinary services in the Amhara region compared to the current study area. Nevertheless, further research is needed on this issue.

Most farmers explained that the stated vaccine price was much lower when compared to the loss of milk production due to FMD. The estimated WTP in MO farming was 64.88 ETB/dose and 23.83 ETB/dose in MF systems, which is relatively high in MO farming. The expected difference in WTP within the MO system was attributed to the comparatively higher income of respondents in this group. However, the calculated WTP in this study significantly surpasses the current price of 15 ETB/dose for the trivalent (O, A, SAT2) vaccine manufactured by the NVI and subsidized by the government. This is consistent with a contingent valuation study for Ethiopia’s Gumboro and Newcastle disease vaccine programs, which disclosed that farmers recognized the value of vaccine programs and were willing to pay for them [20]. When using the estimated WTP for practical applications, it is crucial to consider any possible biases related to it. Willingness to pay estimations frequently shows a tendency to overestimate actual market behavior, according to numerous studies on contingent evaluation [21, 22]. Therefore, it is essential to exercise caution when applying the projected WTP results to real-world situations. Studies across different countries revealed an estimated WTP between 0.4 and 3 USD per dose in addition to the vaccine delivery service fee [2]. In Tanzania, a nearly comparable WTP amount, i.e., 1.84 USD, was reported for the cattle FMD vaccine [23]. Kairu-wanyoike et al. [5] estimated a mean WTP of 3.03 USD for the contagious bovine pleuropneumonia vaccination in the Narok south district of Kenya.

The survey found that approximately 35% of respondents were keen to contribute to FMD control efforts based on the initial bid alone. On the other hand, those unwilling to pay the initial bids constituted a higher percentage (65%) for various reasons, mainly increasing the vaccine price offered. These findings differ significantly from the findings of [20], who found that 57% of the respondents were willing to pay for vaccination against FMD.

None of the sociodemographic variables measured, such as sex, age, education status, and TLU, significantly impacted WTP. Hence, it appears that the main driver for vaccine awareness and WTP is not related to sex, age, TLU, or the education status of the farmer, which is unexpected. Access to livestock extension and benefits-related issues from livestock might play a more significant role in a better WTP [24]. On the other hand, we found that livestock husbandry-related variables such as main livelihood (whether farmers practice MO or MF), keeping animals for profit, and FMD impact perception score served as essential drivers of WTP for the FMD vaccine. The variable main livelihood had a high effect on the mean WTP, indicating that MO respondents are more likely willing to pay. To describe more, the interval regression model computed that the MO respondents had significantly higher WTP than the MF respondents. This was in line with the findings of other studies, as MO farmers often predominantly generate money, as reported in a previous survey [1]. In addition, this finding is in accord with the results of other studies, as those with relatively high incomes often matter for the healthcare expenditure of the household [19]. Hence, the model coefficient at the mean for the main livelihood denoted that the probability that farmers would be willing to pay in MO is 26.0 times higher than that for MF farmers. The difference between the MO and MF systems can be attributed to the income disparity, with the former being wealthier. The farmers practicing the MO cattle farming system predominantly keep exotic cattle breeds or their crosses that are primarily managed under an intensive system, which means they are more affluent and are able to pay for vaccination to prevent any possible loss from FMD diseases, which might affect their income. This is associated with the intention of farmers to rear animals, which is directly related to several benefits gained.

Moreover, MO farmers whose livelihood relies on dairy production would keep more exotic crossbred cattle than local breeds with higher WTP for FMD. Knight-Jones and Rushton, in their studies of the economic impact of FMD, found that the health expenditure for livestock is directly related to income from livestock rearing [2].

Furthermore, farmers who kept animals for profit were more willing to pay (16.0 times) for vaccination than their counterparts who simply rear animals extensively. Those farmers who deliberately raise animals for the sale of animal or animal products such as milk tend to view animal production as a business in which they are eager to invest to mitigate risk and increase the chances of acquiring profits. In their study of FMD outbreak investigation and economic impact assessment in Ethiopia, [25] found that vaccinating animals decreased production losses due to the outbreak and increased animal production and productivity.

As expected, the FMD impact perception score positively influenced farmers’ WTP and was statistically significant at the 4% level. The higher the impact perception score is, the more willing farmers are to contribute to FMD control. Therefore, for every one-unit increase in the farmer’s FMD impact perception score, the WTP for vaccination increased by a factor of 1.40. In line with this, [6] justified that farmers with high-risk perceptions of bovine tuberculosis have a positive attitude toward WTP for its vaccination. It is psychologically rational that farmers with a good perception of disease impact are willing to pay more to prevent disease. However, there was evidence that prior awareness of or having personal knowledge of the disease did not always lead to higher WTP [26].

A separate analysis of MO and MF data to determine drivers of WTP resulted in sales income as a significant variable in the MF system. Sales income affected farmers’ WTP (0.002 times), which is consistent with the theoretical concept of positive income elasticity in that wealthier families purchase more commodities than low-income households [27]. This could be essential for policy implications in Ethiopia, where low-income households often cannot afford proper healthcare for their families and livestock healthcare.

Similarly, owning an exotic type of breed showed a significant association with the farmers’ WTP for FMD vaccination in MO respondents. Respondents who have exotic breeds showed 13.26 times more WTP than those who rear locals and their crosses. This finding closely agrees with the study of WTP for the FMD vaccine reported in northern Ethiopia by [1], which stated that respondents who owned exotic breed cattle and their crosses exhibited a higher WTP compared to those who only owned local cattle breeds. Exotic breeds are valuable investments, motivating farmers to protect them through vaccination. Exotic breeds are more vulnerable to FMD, leading to a greater recognition of the risk and potential economic losses. Those MO farmers often have better financial resources and an understanding of vaccination benefits, allowing them to allocate more funds for disease prevention.

In general, WTP for vaccinations was price-sensitive, as expected. As the offered cost per vaccination increased, the expected WTP decreased from 74 to 11%, which may result in insufficient coverage for cost-effective control. However, we also noticed that wealthier households demanded FMD vaccines even at higher prices. Indeed, the vaccination of more affluent populations would benefit poor people due to herd immunity [28]. Thus, for the full coverage of FMD vaccination in the country, a special fund could be additionally used to subsidize households with lower WTP and support families with lower WTP and those unable to pay.

We sampled households in accessible areas, specifically in central Oromia. As a result, we did not implement strict randomization when selecting districts and villages due to the lack of accessibility to certain rural villages in the study areas. Therefore, our survey sample and results might not reflect all relevant drivers of WTP for livestock farmers throughout the country. Moreover, in applying CVM, a possible source of bias might arise because respondents are not purchasing the vaccine in the practical context as in the hypothetical price [22].

Vaccine traits, a broad concept, were not studied in this choice experiment method. Furthermore, we did not consider herd immunity in the contingent valuation scenario, so our results underestimate the actual value of this particular vaccine. In addition, data of zero protests or those unwilling to pay were not considered in the study, as it was not our objective. Therefore, the above limitations signify possible clues for further research on the above gap. Nevertheless, despite these limitations, the current study tried to identify important factors affecting farmers’ WTP for FMD vaccines.

## Conclusions

The present survey of farmers’ WTP indicated that the mean WTP of respondents was high in both the MF and MO systems, even higher than the current market price in the country. Respondents in the MO system showed higher WTP than those in the MF system. The main livelihood, type of breed, sales income, the animal kept for profit, FMD, impact perception score, and the management system are factors affecting the WTP. The findings of this study show that effective control of FMD at the village level requires coordinated action between the state and the community. Addressing the demand for vaccines, strengthening animal health extension services through educating farmers about the effects of the disease and the importance of vaccines, promoting farming as a business, and further research on WTP and vaccine acceptance were suggested.

### Electronic supplementary material

Below is the link to the electronic supplementary material.

Supplementary Material 1

## Data Availability

Supplimentary material 1 (questionnaire) is uploaded.

## Abbreviations

CI: Confidence Interval
CVM: Contingent Valuation Method
ETB: Ethiopian Birr
FMD: Foot and Mouth Disease
FMDV: Foot and Mouth Disease Virus
MF: Mixed Crop Livestock Farming
MO: Market Oriented
NVI: National Veterinary Institute
SAT: South African Territory
TLU: Tropical Livestock Units
USD: United States Dollar
VIF: Variance Inflation Factor
WTP: Willingness to Pay
