# Supplementary material for: Assessing farmers’ willingness to pay for FMD vaccines and factors influencing payment: a contingent valuation study in central Oromia, Ethiopia
Source: BMC Vet Res. 2024 Jul 15;20:313. doi: 10.1186/s12917-024-04169-7 (PMC11247802; doi:10.1186/s12917-024-04169-7)
Supplement: Supplementary file 1 — Supplementary Material 1 [file 12917_2024_4169_MOESM1_ESM.docx]

**Questionnaire survey for Assessing Farmers' Willingness to Pay for FMD Vaccines and Factors Influencing Payment: A Contingent Valuation Study in Central Oromia, Ethiopia**

- **Do you know foot and mouth disease? If yes, what are typical features of the disease! (if the respondent mention one or more of the following clinical or epidemiological feature of the disease, (s)he is considered as (s)he knows the disease.**
  - 1. Lameness ( foot lesion) and salivation (mouth lesion) in cattle and/ or small ruminants
    2. Foot lesion in cattle and/ or small ruminants that is contagious
    3. Mouth lesion in cattle and/ or small ruminants that is contagious
    4. Lameness or mouth lesion, and blisters (sores)on teats of cows
- **Have you clearly understood the situation stated about the disease? If no, repeat the question until the respondent understood the scenario; but if, Yes, continue with questions below.**
- **Getting to the interview!**
  - 1. Are you willing to pay the proposed price i.e.12 **ETB/Dose** for the vaccine explained above?

1. Yes B. No C. Undetermined
2. If yes to question **1** above, what if the vaccine price is **18 ETB/Dose** (50 %) increase in the original price.)
3. Yes B. No C. Undetermined
4. If no to question **1** above what if the vaccine price is **6 ETB/Dose** (50 % decrease in the original price.
5. Yes B. No C. Undetermined

**Part III. Socio demographic variables**

1. Respondents (household head). Name____________________________­­­­­­­­­­­­­­­­­­­­­­­­­­­­­­­­­­­­­­­­­­___Sex _________Age _______Education status_________________ Household size ____________Main livelihood ____­­­___________________________________
2. Contribution of livestock to livelihood a. Main livelihood b. Supplementary

- **Economic variables**

1. Livestock owned: #cattle, ___#sheep, ____#goat,_____ #donkey,____ #horse, ____#mule, ____#poultry____.
2. No. cattle sold or income from the sale of cattle in the last one year __________
3. Quantity of milk sold or income from the sale of milk in the last one year _____
4. Quintal of crop harvested in the previous year ___________
5. Other agricultural related income in the previous year _____________
6. Annual income for off farm activities­______________

- **Husbandry and Disease control practices**

1. Type of production system in the area
2. Pastoral B. Agro-pastoral C. Sedentary
3. Management/husbandry system practiced
4. Intensive B. Semi-intensive C. Extensive
5. The breeds of cattle owned
6. Local breeds **(Arsi breed, Ambo breed, Borena breed, Horro breed**)
7. Crossed breeds (**Non-descript crossed breeds**)
8. Pure Exotic breed **(Holstein Friesian, Jersey breed)**
9. Involvement in market-oriented production like fatting and milk sale
10. Yes B. No
11. Frequency of use of modern veterinary service when faced with animal health problem
12. Always B. Sometimes C. Never
13. Traditional medicine and practices as source of animal health service
14. Main B. Supplementary C. Not at all
15. What are barriers to FMD vaccination?
16. High cost B. Unavailability C. Lack of knowledge on benefits of the vaccine D. perceiving the disease as it is not important.
17. Do you vaccinate your cattle for FMD annually? A. Yes B. No

- **Knowledge/Perception on Impact of FMD**

1. The frequency of FMD occurrence in your area
2. Very low B. Low C. Moderate D. High E. Very high
3. The impact of FMD on Milk production
4. Very low B. Low C. Moderate D. High E. Very high
5. The impact of FMD on draught power is
6. Very low B. Low C. Moderate D. High E. Very high
7. The impact of FMD in terms of Mortality and body condition.
8. Very low B. Low C. Moderate D. High E. very high
9. Cost of treatment of FMD ill animals
10. Very low B. Low C. Moderate D. High E. very high

- **Knowledge/ perception about livestock vaccines**

1. Vaccine are very useful to prevent disease
2. Yes B. No C. Don’t know
3. Some vaccines are better than others
4. Agree B. Dis-agree C. Neutral
5. Vaccination protects others
6. Agree B. Disagree C. Moderately agree
7. Do you get information about vaccines?
8. Yes B. No
9. If yes to question **28**, what is the source of information?
10. Media B. Neighbors C. Friends D. vets
11. Vaccination do not cure diseased animals
12. Yes B. No C. Don’t know
13. One vaccine is used only for one disease
14. Yes B. No C. Don’t know
15. Most Vaccines give protection only for a limited time
16. Yes B. No C. Don’t know
